# New progress in immunobiology and transplantation research

**DOI:** 10.4103/2321-3868.126080

**Published:** 2014-01-26

**Authors:** Xian C. Li

**Affiliations:** Department of Surgery, Transplant Immunology Program, Houston, Methodist Hospital, Texas Medical Center, Houston, Texas 77030 USA

In this special issue of Burns & Trauma, we focus on transplant immunology, highlighting the pressing issues in the field and emerging strategies in resolving these issues, especially in the area of tolerance induction. The ultimate goal is to achieve transplant tolerance, a state of stable transplant survival without lifelong immunosuppression.

This topic is a timely one, as solid organ transplantation has clearly come of age, and within a relatively short period of time, transplantation developed from just an exploratory procedure to a preferred treatment of choice for end-stage organ failure. From a clinical perspective, the short-term transplant survival has been excellent now, with 1-year survival greater than 90% for most organ transplants in the clinic. This is mainly due to improvement in immunosuppressive protocols that most effectively control acute rejection. This remarkable accomplishment has resulted in significant advances in many other fronts in clinical medicine, including vascularized composite tissue allotransplantation (VCA) for trauma patients whose injuries are just beyond repair by conventional surgeries. The current experience with hand and face transplantation in the world for those with debilitating and dysfiguring injuries has generated tremendous enthusiasm in that transplantation of multiple different tissues and organs together as a single unit (like a hand) can offer a therapeutic option for some patients that otherwise have no choice of treatment at all.

However, VCA for burn and trauma patients creates both challenges and opportunities. Composite tissue grafts (e.g., limbs and face transplants), like most transplanted organs, are subject to immune attacks by the recipients. However, unlike other solid organ transplantation, composite tissue transplants are composed of multiple tissues with different functional properties, including skin, muscle, bone, and nerves. These tissues also have different immunological features. Therefore, it is important to elucidate the key features unique to individual tissues and how such different tissues interface with the recipient’s immune system. To date, patients with a composite tissue transplant have to take immunosuppression drugs for life, the risk and the benefit of VCA versus side effects of long-term immunosuppression need to be carefully considered. Key areas of future investigation should include fundamental mechanisms of composite tissue rejection, new protocols aimed at producing transplant survival without lifelong immunosuppression drugs, and innovative ways to minimize tissue injury and foster tissue regeneration. Studies in areas may bring new advances to transplant medicine that will benefit patients who suffer from disfiguring trauma and burns. For this topic, Tullius *et al.*, (next issue) will summarize the current status of VCA, highlighting new development and challenges in the field. Kloc *et al.*, discussed the mechanisms of chronic allograft rejection, the cell types involved, and new emerging therapies to treat or prevent chronic graft loss. [1] One of the key features of chronic rejection is the slow and progressive destruction of graft vasculature, which is a major cause of graft loss in clinical transplantation, and because of this, there is an urgent need now to develop new therapies to prevent damage of graft vasculature. Chen *et al.*, focused on the role of cytokines in the allograft response, pointing to the facts that cytokines can be proinflammatory favoring transplant rejection or anti-inflammatory facilitating tolerance. Thus, cytokines may be a powerful tool with which to selectively and specifically modulate the immune responses.[2] Dai *et al.*, highlighted the importance of regulatory cells in the induction of transplant tolerance, the diversity of immune cells with regulatory properties, and emerging approaches to therapeutically target regulatory cells in the clinic.[3] Moreover, Zhou *et al.*, described an interleukin (IL)-10 related T cell proliferation inhibition in mixed lymphocyte reaction induced by multiple alloantigens, providing new evidence for the mechanism of T cell intraclonal competition.[4] Hopefully, these articles will provide the readers with a focused glimpse of several rapidly developing areas of VCA as well as challenges that are associated with it, especially in the area of reprogramming the immune system to create lasting tolerance.

**Table 1:** 

Access this article online	
**Quick Response Code:**	**Website:** www.burnstrauma.com
	**DOI:** 10.4103/2321-3868.126080
